# Prevalence of Arthritis and Arthritis-Attributable Activity Limitation by Urban-Rural County Classification — United States, 2015

**DOI:** 10.15585/mmwr.mm6620a2

**Published:** 2017-05-26

**Authors:** Michael A. Boring, Jennifer M. Hootman, Yong Liu, Kristina A. Theis, Louise B. Murphy, Kamil E. Barbour, Charles G. Helmick, Terry J. Brady, Janet B. Croft

**Affiliations:** 1 Division of Population Health, National Center for Chronic Disease Prevention and Health Promotion, CDC.

Rural populations in the United States have well documented health disparities, including higher prevalences of chronic health conditions (*1*,*2*). Doctor-diagnosed arthritis is one of the most prevalent health conditions (22.7%) in the United States, affecting approximately 54.4 million adults (*3*). The impact of arthritis is considerable: an estimated 23.7 million adults have arthritis-attributable activity limitation (AAAL). The age-standardized prevalence of AAAL increased nearly 20% from 2002 to 2015 (*3*). Arthritis prevalence varies widely by state (range = 19%–36%) and county (range = 16%–39%) (*4*). Despite what is known about arthritis prevalence at the national, state, and county levels and the substantial impact of arthritis, little is known about the prevalence of arthritis and AAAL across urban-rural areas overall and among selected subgroups. To estimate the prevalence of arthritis and AAAL by urban-rural categories CDC analyzed data from the 2015 Behavioral Risk Factor Surveillance System (BRFSS). The unadjusted prevalence of arthritis in the most rural areas was 31.8% (95% confidence intervals [CI] = 31.0%–32.5%) and in the most urban, was 20.5% (95% CI = 20.1%–21.0%). The unadjusted AAAL prevalence among adults with arthritis was 55.3% in the most rural areas and 49.7% in the most urban. Approximately 1 in 3 adults in the most rural areas have arthritis and over half of these adults have AAAL. Wider use of evidence-based interventions including physical activity and self-management education in rural areas might help reduce the impact of arthritis and AAAL.

BRFSS is an ongoing, state-based, random-digit–dialed landline and cellphone survey of the noninstitutionalized adult population aged ≥18 years of the 50 states, the District of Columbia (DC), and the U.S. territories. BRFSS, designed to provide national and state-level estimates, collects data on health-related risk behaviors and chronic health conditions. Among the 2015 BRFSS respondents surveyed in the 50 states and DC, complete information on age, county, and arthritis diagnosis was available for 426,361 (98.2%). The median combined response rate for the 2015 BRFSS was 47.2% and ranged from 33.9% in California to 61.1% in Utah.* Respondents were classified as having arthritis if they answered “yes” to the question, “Have you ever been told by a doctor or other health professional that you have some form of arthritis, rheumatoid arthritis, gout, lupus, or fibromyalgia?” Among adults with arthritis, AAAL was identified by a “yes” response to the question, “Are you now limited in any way in any of your usual activities because of arthritis or joint symptoms?”

Counties were classified into six urban-rural categories using the National Center for Health Statistics 2013 Urban-Rural Classification Scheme for Counties,^†^ based on 2010 U.S. Census data and the 2013 Office of Management and Budget designations of metropolitan statistical areas, micropolitan statistical areas, and noncore areas. The county classification categories from most urban to most rural are 1) large central metropolitan (city); 2) large fringe metropolitan (suburb); 3) medium metropolitan; 4) small metropolitan; 5) micropolitan; and 6) noncore (rural).

Unadjusted overall, age-specific, and age-standardized prevalence with CIs were estimated for arthritis and AAAL by urban-rural categories. Age-standardized prevalence by urban-rural categories was further stratified by selected demographic (sex, race/ethnicity, highest education level, and employment status) and health (body mass index, leisure time physical activity, self-rated health, disability, and smoking status) characteristics. Estimates were age-standardized to the 2000 U.S. standard population aged ≥18 years using three age groups (18–44 years, 45–64 years, and ≥65 years).^§^ All analyses accounted for the complex sampling design of the survey, with sampling weights created using raking methodology. This methodology allows incorporation of many demographic variables into the weighting process, including telephone source, which makes the sample more representative of the population and reduces the potential for bias. Statistical significance was determined using t-tests at α = 0.05 with the most rural (noncore) category as the reference group.

In the most rural areas (noncore) nearly 1 in 3 adults (unadjusted prevalence 31.8%) reported having doctor-diagnosed arthritis (Table 1). Age-specific prevalence was higher in older age groups and in rural areas. In age-standardized analyses, the prevalence of arthritis was lower among adults living in the most urban areas (20.0%; 95% CI = 19.6%–20.5%), and higher among adults living in the most rural areas (26.9%; 95% CI = 26.2%–27.5%) (Table 1) (Figure). Age-standardized arthritis prevalence was higher in rural areas among most subgroups studied. Across all urban-rural categories, arthritis prevalence followed previously reported patterns for U.S. adults: higher prevalence among women, older adults, smokers, adults with less education, adults who are less physically active, or adults with higher body mass index. Arthritis prevalence was ≥50% among adults aged ≥65 years across all urban-rural categories (50%–55%), adults unable to work because of disability in all but the most urban categories (50%–57%), and adults reporting any functional disability in the most rural category (50%) (Table 1).

**TABLE 1:** Prevalence of doctor-diagnosed arthritis (crude and age-standardized) among U.S. adults aged ≥18 years, by urban-rural status and selected characteristics — Behavioral Risk Factor Surveillance System, 2015*

Characteristics	Large metro center (city)	Large fringe metro (suburb)	Medium metro	Small metro	Micropolitan	Noncore (rural)
No. of respondents	69,362	81,703	92,484	57,348	65,004	60,460
No. with arthritis	20,333	26,651	31,069	20,000	23,703	22,931
**Prevalence**	**% (95% CI)**	**% (95% CI)**	**% (95% CI)**	**% (95% CI)**	**% (95% CI)**	**% (95% CI)**
Unadjusted	20.5 (20.1–21.0)	24.3 (23.8–24.8)	25.9 (25.4–26.4)	27.2 (26.6–27.8)	29.6 (28.9–30.2)	31.8 (31.0–32.5)
Age-standardized^†^	20.0 (19.6–20.5)	22.0 (21.6–22.5)	23.7 (23.3–24.2)	24.6 (24.1–25.2)	26.1 (25.5–26.7)	26.9 (26.2–27.5)
**Age group (yrs)**						
18–44	6.0 (5.6–6.5)	7.7 (7.2–8.2)	9.0 (8.5–9.6)	9.1 (8.4–9.9)	11.1 (10.3–12.0)	11.0 (10.2–11.9)
45–64	28.0 (27.0–28.9)	30.8 (30.0–31.7)	33.8 (33.0–34.7)	36.3 (35.1–37.4)	37.0 (35.9–38.1)	39.2 (38.0–40.4)
≥65	49.7 (48.3–51.0)	51.2 (50.1–52.2)	51.9 (50.9–52.8)	52.7 (51.5–54.0)	53.8 (52.6–55.0)	54.7 (53.4–55.9)
**Sex^†^**						
Male	16.8 (16.1–17.4)	18.9 (18.3–19.4)	20.7 (20.1–21.3)	21.7 (20.9–22.5)	23.0 (22.2–23.9)	23.8 (23.0–24.7)
Female	22.9 (22.3–23.5)	24.9 (24.3–25.5)	26.6 (26.0–27.2)	27.5 (26.7–28.3)	29.2 (28.3–30.0)	29.8 (28.9–30.7)
**Race/Ethnicity^†^**						
White, non-Hispanic	21.4 (20.8–22.0)	23.4 (22.9–23.9)	25.0 (24.5–25.6)	25.4 (24.7–26.1)	27.0 (26.3–27.8)	27.7 (27.0–28.4)
Black, non-Hispanic	22.9 (21.7–24.1)	22.6 (21.3–24.1)	24.5 (23.2–25.8)	26.0 (23.8–28.3)	24.9 (23.1–26.8)	25.8 (23.8–27.9)
Hispanic	18.1 (17.0–19.3)	16.8 (15.3–18.4)	18.2 (17.0–19.5)	17.7 (15.8–19.8)	17.3 (15.1–19.6)	16.6 (14.0–19.6)
American Indian/ Alaska Native	32.1 (25.3–39.8)	30.0 (24.1–36.7)	31.9 (27.8–36.2)	33.1 (28.2–38.3)	30.6 (26.7–34.8)	26.3 (23.2–29.7)
Asian	11.4 (9.4–13.6)	11.5 (9.2–14.2)	15.2 (12.0–19.1)	11.8 (8.8–15.6)	9.9 (7.2–13.3)	23.5 (16.6–32.3)
Native Hawaiian/ Pacific Islander	29.5 (21.8–38.7)	21.5 (13.8–31.8)	16.1 (10.2–24.6)	29.8 (18.3–44.6)	14.4 (8.3–23.8)	UR^§^
Multiracial, non-Hispanic	24.6 (21.2–28.3)	28.4 (24.9–32.1)	30.2 (27.4–33.1)	30.6 (26.1–35.5)	35.4 (31.4–39.5)	35.1 (29.8–40.7)
Others, non-Hispanic	15.2 (11.4–19.9)	20.8 (15.9–26.6)	31.3 (24.1–39.6)	28.8 (22.3–36.4)	23.0 (15.6–32.4)	29.6 (17.1–46.1)
**Education^†^**						
Less than HS	21.8 (20.4–23.2)	25.9 (24.2–27.6)	26.8 (25.4–28.3)	28.5 (26.5–30.5)	31.4 (29.2–33.6)	31.9 (29.9–33.9)
HS or equivalent	21.7 (20.8–22.6)	23.7 (22.9–24.5)	24.9 (24.1–25.7)	26.4 (25.4–27.5)	25.9 (25.0–26.9)	27.6 (26.6–28.5)
Some college	21.9 (21.1–22.8)	23.7 (22.9–24.5)	24.8 (24.1–25.6)	24.9 (23.9–25.9)	26.7 (25.7–27.7)	26.5 (25.4–27.6)
College and above	15.9 (15.3–16.5)	17.8 (17.2–18.3)	19.3 (18.7–19.9)	19.3 (18.5–20.1)	20.8 (19.9–21.7)	20.3 (19.2–21.4)
**Employment^†^**						
Employed/Self-employed	15.6 (15.0–16.3)	17.7 (17.1–18.3)	19.2 (18.5–19.8)	19.0 (18.2–19.8)	20.8 (20.0–21.6)	20.2 (19.3–21.0)
Unemployed	20.0 (18.0–22.1)	21.9 (19.8–24.0)	22.9 (20.7–25.3)	26.9 (23.9–30.1)	26.5 (23.3–30.0)	27.3 (24.1–30.8)
Retired	28.6 (23.0–35.0)	28.9 (23.5–34.9)	38.9 (29.7–48.9)	40.4 (30.1–51.7)	47.0 (31.9–62.7)	39.1 (25.0–55.3)
Unable to work because of disability	42.3 (39.6–45.2)	49.8 (46.6–53.0)	51.8 (49.1–54.4)	56.4 (52.6–60.1)	54.8 (51.3–58.3)	56.7 (53.2–60.2)
Other (student/ homemaker)	19.6 (18.0–21.3)	22.3 (20.9–23.8)	22.8 (21.5–24.1)	22.7 (20.9–24.7)	24.5 (22.4–26.8)	24.5 (22.6–26.5)
**Health characteristics**						
**Body mass index (kg/m^2^)^†^**						
<25.0 (under/normal weight)	16.4 (15.7–17.1)	17.7 (17.0–18.4)	19.1 (18.5–19.8)	20.3 (19.3–21.3)	21.3 (20.3–22.4)	22.3 (21.3–23.4)
25.0–29.9 (overweight)	18.7 (17.9–19.4)	20.6 (19.9–21.3)	22.2 (21.5–23.0)	22.8 (21.8–23.8)	23.4 (22.5–24.4)	24.6 (23.4–25.7)
≥30 (obese)	27.4 (26.5–28.4)	30.1 (29.1–31.1)	31.0 (30.1–32.0)	31.3 (30.2–32.4)	34.3 (33.1–35.6)	33.3 (32.2–34.5)
**Smoking status^†^**						
Current smoker	25.2 (23.8–26.6)	28.6 (27.3–30.1)	29.3 (28.2–30.5)	30.1 (28.6–31.7)	32.0 (30.4–33.6)	34.1 (32.5–35.7)
Former smoker	24.1 (23.1–25.2)	25.8 (24.8–26.7)	27.0 (25.9–28.0)	27.3 (26.0–28.6)	29.8 (28.4–31.3)	29.8 (28.4–31.3)
Never smoker	17.5 (16.9–18.0)	18.8 (18.3–19.3)	20.8 (20.3–21.3)	21.5 (20.9–22.2)	22.0 (21.3–22.8)	22.4 (21.7–23.2)
**Physical activity (aerobic)** ^†,¶^						
Active	18.2 (17.6–18.9)	20.4 (19.8–20.9)	21.4 (20.8–22.0)	22.3 (21.5–23.1)	23.4 (22.5–24.2)	24.5 (23.6–25.5)
Insufficiently active	19.1 (18.1–20.2)	21.5 (20.5–22.5)	23.6 (22.6–24.6)	24.3 (22.9–25.7)	25.3 (24.0–26.6)	25.3 (23.8–26.7)
Inactive	24.8 (23.8–25.8)	26.8 (25.9–27.8)	29.2 (28.2–30.2)	29.6 (28.3–31.0)	32.2 (30.8–33.6)	31.3 (30.0–32.5)
**Self-rated health^†^**						
Excellent/Very good	13.8 (13.2–14.3)	15.5 (15.0–16.0)	16.3 (15.8–16.8)	16.3 (15.7–16.9)	17.1 (16.4–17.8)	16.6 (15.9–17.3)
Good	21.0 (20.2–21.9)	24.2 (23.4–25.0)	25.2 (24.4–26.0)	26.4 (25.4–27.5)	27.2 (26.1–28.3)	27.5 (26.3–28.6)
Fair/Poor	34.2 (32.8–35.7)	40.9 (39.2–42.7)	41.2 (39.8–42.6)	43.8 (41.8–45.9)	46.2 (44.1–48.4)	47.9 (45.8–50.1)
**Functionally disabled**^†,^**						
Yes	37.8 (36.5–39.1)	43.1 (41.6–44.5)	44.2 (42.9–45.5)	46.1 (44.3–47.9)	47.9 (46.1–49.7)	49.8 (47.9–51.8)
No	14.8 (14.3–15.2)	16.9 (16.5–17.3)	17.5 (17.1–17.9)	17.8 (17.3–18.4)	18.7 (18.1–19.3)	18.6 (18.0–19.2)

**Abbreviations:** CI = confidence interval; HS = high school; UR = unreliable.

* Estimates are weighted and account for the complex sampling design.

^†^ Estimates age-standardized to the 2000 U.S standard population aged ≥18 years using three groups (18–44 years, 45–64 years, ≥65 years).

^§^ Estimates are unreliable and are suppressed (sample size <50 or relative standard error >30%).

^¶^ Respondents were classified as active if they reported ≥150 minutes of moderate intensity leisure time aerobic physical activity per week, insufficiently active if they reported 1–149 minutes, and inactive if they reported 0 minutes. Reported vigorous intensity physical activity minutes were counted double and added to moderate intensity physical activity minutes.

** Respondents were classified as functionally disabled if they answered yes to any of the following five questions: “Because of a physical, mental, or emotional condition, do you have serious difficulty concentrating, remembering, or making decisions?”; “Do you have serious difficulty walking or climbing stairs?”; “Are you blind or do you have serious difficulty seeing, even when wearing glasses?”; “Do you have difficulty dressing or bathing?”; “Because of a physical, mental, or emotional condition, do you have difficulty doing errands alone such as visiting a doctor’s office or shopping?”

**FIGURE:**
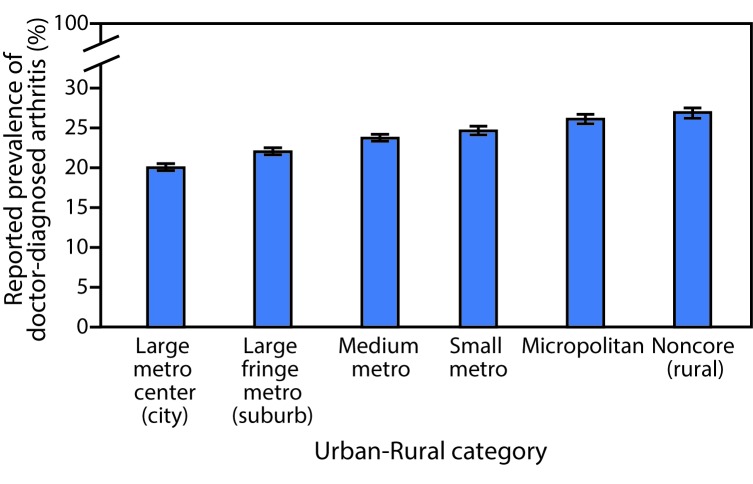
Age-standardized arthritis prevalence, by urban-rural categories — Behavioral Risk Factor Surveillance Survey, United States, 2015

AAAL affected about half of adults with arthritis in all urban-rural categories; unadjusted overall prevalence ranged from 47.8% to 55.3% (Table 2). Age-specific prevalence of AAAL was higher in rural areas and among persons aged 45–64 years in all areas. In age-standardized analyses, the overall prevalence of AAAL was lower among adults in the most urban category (47.1%, 95% CI = 44.9%–49.3%), and higher in the most rural (56.9%, 95% CI = 54.6%–59.2%) (Table 2). Across the majority of health characteristic and demographic subgroups studied, higher prevalences of AAAL were found in the most rural (noncore) category (Table 2).

**TABLE 2:** Prevalence (crude and age-standardized) of arthritis-attributable activity limitation (AAAL) among adults aged ≥18 years with doctor-diagnosed arthritis, by urban-rural status and selected characteristics — Behavioral Risk Factor Surveillance System, 2015*

Characteristics	Large metro center (city)	Large fringe metro (suburb)	Medium metro	Small metro	Micropolitan	Noncore (rural)
No. with arthritis^†^	18,228	24,213	28,377	18,353	21,803	21,092
No. with AAAL	9,005	11,550	14,026	9,130	11,037	10,905
**Prevalence**	**% (95% CI)**	**% (95% CI)**	**% (95% CI)**	**% (95% CI)**	**% (95% CI)**	**% (95% CI)**
Unadjusted	49.7 (48.3–51.1)	47.8 (46.7–48.9)	50.2 (49.2–51.3)	50.9 (49.6–52.2)	51.7 (50.4–52.9)	55.3 (54.0–56.6)
Age-standardized^§^	47.1 (44.9–49.3)	48.6 (46.6–50.6)	49.7 (47.8–51.5)	50.6 (48.0–53.1)	52.4 (50.1–54.7)	56.9 (54.6–59.2)
**Age group (yrs)**						
18–44	42.9 (39.0–46.9)	49.0 (45.4–52.6)	47.9 (44.6–51.3)	48.8 (44.2–53.4)	52.3 (48.1–56.5)	57.4 (53.1–61.5)
45–64	54.6 (52.5–56.6)	50.2 (48.5–51.9)	54.8 (53.2–56.4)	56.2 (54.2–58.2)	55.4 (53.5–57.3)	60.4 (58.5–62.3)
≥65	47.0 (45.0–49.0)	44.8 (43.3–46.4)	46.1 (44.7–47.5)	46.1 (44.4–47.9)	47.6 (45.9–49.4)	49.3 (47.5–51.1)
**Sex^†^**						
Male	41.3 (37.6–45.0)	45.9 (42.6–49.2)	45.3 (42.5–48.3)	46.0 (42.3–49.8)	48.6 (45.0–52.2)	53.5 (49.7–57.4)
Female	51.1 (48.4–53.8)	50.5 (47.9–53.0)	52.9 (50.5–55.2)	54.3 (50.9–57.7)	55.2 (52.2–58.2)	59.3 (56.4–62.1)
**Race/Ethnicity** ^§^						
White, non-Hispanic	48.3 (45.2–51.3)	48.6 (46.4–50.8)	47.4 (45.2–49.6)	49.6 (46.8–52.4)	51.5 (48.9–54.1)	56.5 (53.9–59.1)
Black, non-Hispanic	46.2 (41.6–50.8)	48.2 (42.1–54.2)	55.0 (49.1–60.8)	52.6 (43.1–62.0)	56.6 (48.6–64.2)	57.9 (49.2–66.1)
Hispanic	43.2 (38.3–48.1)	52.1 (44.4–59.7)	58.8 (53.2–64.3)	50.7 (40.4–60.8)	49.7 (40.4–59.1)	58.2 (44.3–71.0)
American Indian/ Alaska Native	64.4 (49.7–76.8)	63.3 (47.0–77.0)	57.9 (46.0–69.0)	64.1 (46.2–78.8)	68.2 (54.1–79.5)	59.4 (49.7–68.4)
Asian	40.6 (28.4–54.1)	38.1 (25.2–53.0)	45.1 (25.7–66.0)	UR^¶^	49.6 (29.6–69.8)	UR^¶^
Native Hawaiian/ Pacific Islander	UR^¶^	UR^¶^	UR^¶^	UR^¶^	UR^¶^	UR^¶^
Multiracial, non-Hispanic	59.4 (48.5–69.5)	59.7 (45.4–72.5)	54.9 (46.8–62.6)	68.0 (54.2–79.2)	67.1 (54.3–77.8)	59.2 (44.6–72.4)
Others, non-Hispanic	63.5 (37.4–83.4)	55.0 (35.6–73.0)	76.4 (63.9–85.5)	UR^¶^	27.6 (20.6–35.9)	74.5 (61.9–84.0)
**Education** ^§^						
Less than HS	54.6 (47.5–61.4)	54.8 (47.8–61.7)	65.6 (60.0–70.8)	62.9 (55.0–70.2)	62.3 (55.0–69.2)	69.5 (63.7–74.7)
HS or equivalent	50.8 (46.1–55.4)	48.9 (44.9–53.0)	48.4 (44.7–52.1)	50.0 (45.6–54.3)	51.3 (47.4–55.1)	55.1 (51.1–58.9)
Some college	45.8 (42.2–49.4)	52.1 (48.7–55.4)	49.8 (46.8–52.9)	50.9 (46.7–55.1)	52.3 (48.6–55.9)	54.3 (50.5–58.1)
College graduate	40.4 (36.9–43.9)	40.5 (37.3–43.9)	39.6 (36.7–42.5)	40.1 (36.0–44.3)	41.9 (37.3–46.6)	48.2 (42.8–53.6)
**Employment** ^§^						
Employed/Self-employed	36.1 (33.3–39.1)	39.5 (37.0–42.2)	38.1 (35.7–40.5)	37.0 (33.9–40.2)	37.9 (34.9–40.9)	42.2 (38.9–45.5)
Unemployed	51.9 (43.8–59.8)	56.9 (49.3–64.2)	64.3 (58.2–70.0)	53.2 (44.7–61.5)	64.8 (57.0–71.9)	60.9 (52.2–69.0)
Retired	51.8 (28.2–74.7)	57.5 (38.9–74.1)	61.6 (44.6–76.2)	65.6 (48.4–79.6)	74.9 (72.1–77.5)	UR^¶^
Unable to work because of disability	83.7 (79.2–87.4)	81.0 (76.1–85.1)	77.6 (72.8–81.7)	81.7 (74.9–87.0)	83.2 (78.7–86.9)	84.3 (80.9–87.2)
Other (student/ homemaker)	47.7 (41.7–53.7)	47.0 (41.2–53.0)	47.0 (41.7–52.4)	49.1 (41.2–57.1)	48.6 (41.6–55.7)	51.6 (44.7–58.4)
**Health characteristics**						
**Body mass index (kg/m^2^)** ^§^						
<25.0 (under/normal weight)	44.4 (40.5–48.4)	45.6 (41.8–49.4)	46.7 (43.0–50.4)	50.4 (45.4–55.5)	51.5 (46.5–56.3)	56.2 (51.7–60.5)
25.0–29.9 (overweight)	42.2 (37.9–46.6)	47.2 (43.5–51.0)	42.9 (39.6–46.4)	48.2 (43.3–53.1)	45.9 (41.6–50.1)	55.4 (50.4–60.3)
≥30.0 (obese)	52.6 (49.0–56.2)	51.7 (48.4–55.0)	56.9 (54.0–59.7)	52.2 (48.5–55.8)	57.4 (53.9–60.8)	58.9 (55.4–62.3)
**Smoking status** ^§^						
Current smoker	58.6 (54.0–63.1)	58.3 (54.5–62.0)	56.4 (53.1–59.6)	61.8 (57.5–66.0)	60.6 (56.9–64.2)	62.7 (58.9–66.4)
Former smoker	44.9 (40.3–49.5)	48.1 (44.1–52.2)	50.4 (46.3–54.5)	47.6 (42.7–52.6)	51.5 (46.6–56.3)	57.5 (52.6–62.2)
Never smoker	42.8 (39.9–45.7)	43.4 (40.6–46.3)	45.3 (42.5–48.1)	43.2 (39.4–47.1)	46.3 (42.5–50.1)	51.2 (47.4–55.0)
**Physical activity (aerobic)**^§,^**						
Activeⱡ	41.7 (38.7–44.9)	43.4 (40.4–46.4)	42.8 (40.1–45.5)	44.9 (41.2–48.7)	45.0 (41.5–48.6)	52.0 (48.3–55.8)
Insufficiently active	44.6 (39.9–49.3)	49.6 (45.4–53.7)	50.8 (46.9–54.7)	49.2 (43.6–54.8)	51.3 (46.3–56.2)	53.9 (48.5–59.3)
Inactive	55.3 (51.0–59.5)	54.5 (50.7–58.3)	58.9 (55.4–62.3)	58.7 (54.2–63.0)	61.2 (57.4–64.8)	64.3 (60.7–67.8)
**Self-rated health** ^§^						
Excellent/Very good	30.4 (27.1–33.8)	32.4 (29.3–35.7)	30.5 (27.6–33.6)	30.0 (26.5–33.8)	31.8 (28.1–35.9)	34.9 (30.8–39.2)
Good	42.5 (38.8–46.3)	48.4 (44.8–51.9)	49.1 (45.8–52.4)	46.8 (42.4–51.3)	46.9 (43.1–50.8)	51.9 (47.7–56.0)
Fair/Poor	68.4 (63.9–72.6)	67.6 (63.9–71.1)	69.7 (66.5–72.6)	71.8 (67.7–75.6)	73.4 (69.9–76.6)	75.1 (71.8–78.2)
**Functionally disabled** ^§,††^						
Yes	70.3 (66.7–73.7)	74.9 (72.1–77.6)	73.8 (71.1–76.3)	72.9 (69.0–76.5)	75.6 (72.8–78.1)	79.1 (76.4–81.5)
No	28.7 (26.0–31.5)	30.1 (27.7–32.5)	28.9 (26.6–31.3)	28.5 (25.5–31.7)	28.9 (26.3–31.6)	32.1 (28.9–35.4)

**Abbreviations:** CI = confidence interval; HS = high school, UR = unreliable.

* Estimates are weighted and account for the complex sampling design.

^†^ Analysis for AAAL prevalence excluded respondents where AAAL could not be ascertained (Don’t know/Not sure, Refused, or missing).

^§^ Estimates are age-standardized to the 2000 U.S standard population aged ≥18 years using three groups (18–44 years, 45–64 years, ≥65 years).

^¶^ Estimates are unreliable and are suppressed (sample size <50 or relative standard error >30%).

** Respondents were classified as active if they reported ≥150 minutes of moderate intensity leisure time aerobic physical activity per week, insufficiently active if they reported 1–149 minutes, and inactive if they reported 0 minutes. Reported vigorous intensity physical activity minutes were counted double and added to moderate intensity physical activity minutes.

^††^ Respondents were classified as functionally disabled if they answered yes to any of the following five questions: “Because of a physical, mental, or emotional condition, do you have serious difficulty concentrating, remembering, or making decisions?”; “Do you have serious difficulty walking or climbing stairs?”; “Are you blind or do you have serious difficulty seeing, even when wearing glasses?”; “Do you have difficulty dressing or bathing?”; “Because of a physical, mental, or emotional condition, do you have difficulty doing errands alone such as visiting a doctor’s office or shopping?”

## Discussion

In 2015, rural U.S. residents experienced a high prevalence and negative impact of arthritis. In the most rural areas, nearly 1 in 3 adults had arthritis and among adults with arthritis, approximately half reported being limited by arthritis. Prevalence of arthritis and AAAL was particularly high among rural residents with a functional or work disability. Rural populations might have higher prevalence of arthritis and AAAL because of recognized rural risk factors including older age, obesity, and lower socioeconomic status (*1*,*2*).

Several evidence-based physical activity and self-management education programs^¶^ can help decrease the impact of AAAL by reducing pain and improving function, mood, and quality of life (*5*). Many of these programs are offered in small groups, with limited availability in rural areas. For example, a national implementation of one self-management education program, the Chronic Disease Self-Management Program, reached less than 25% of all U.S. rural areas (*6*). However, engaging in proven self-directed versions of these programs (e.g., Walk with Ease, The Arthritis Toolkit) could represent inexpensive and accessible options. Community organizations already serving rural populations, including churches, county extension agents, veterans’ service organizations, health care clinics, and community centers might be able to collaborate to make the small-group versions of these low-cost programs more available.

Physical activity is a proven intervention for managing arthritis and reducing the impact of arthritis-attributable activity limitations (*7*). Walking is a low impact, accessible activity proven to reduce pain and improve quality of life for adults with arthritis (*8*). In micropolitan areas, an important environmental barrier to walking is limited pedestrian infrastructure including long distances between destinations and lack of sidewalks (*9*). Changes in land use (e.g., parks and trails), destination locations (e.g., coffee shops, post offices) and transportation infrastructure (e.g., presence of sidewalks and crosswalks, light signals) have been associated with environments that facilitate increased walking in many geographic areas and some of these components might also apply in smaller rural areas (*9*). These changes could provide an environment that facilitates walking among rural residents.

Health care providers can help their patients manage their arthritis by recommending physical activity and self-management education programs. Adults with arthritis are more likely to attend a self-management education program when it is recommended by a health care provider.**

The prevalence of arthritis and AAAL among adults with work and functional disabilities were substantial; at least four of five rural residents with a functional or work disability had AAAL. Persons of all ages with work disabilities could benefit from Job Accommodation Network (JAN) services.^††^ JAN is a free federal resource that provides job accommodation information, links persons needing accommodation and employers to legal advice, and facilitates contact with additional state-specific and other employment resources, including state-based vocational rehabilitation and job retraining resources.

The findings in this study are subject to at least four limitations. First, arthritis is self-reported and the diagnosis was not confirmed by a health care professional; however, this case definition has been validated for public health surveillance (*10*). Second, the health-related behaviors are self-reported and therefore subject to social desirability bias. Third, findings are generalizable only to the civilian, non-institutional population, as the survey does not include adults who live in long-term care facilities, prisons, and other institutions. Finally, low response rates can result in nonresponse bias and response rates by urban-rural classifications are not reported. However, the use of raking weighting methodology adjusts for nonresponse bias.^§§^

Despite these limitations, this study has multiple strengths. BRFSS collects information on a wide range of demographics, chronic conditions and health behaviors. Additionally, the large sample size allowed calculation of statistically precise estimates across all six urban-rural classifications overall and by subgroups.

The higher prevalence of arthritis and AAAL among rural U.S. residents highlights the need for evidence-based intervention approaches such as physical activity, self-management education, and vocational rehabilitation programs. Health care providers and community organizations that serve rural residents can help adults with arthritis in rural areas increase access to and participation in interventions that are proven to reduce pain, improve function and quality of life, and maintain workforce participation.

**Summary**

**What is already known about this topic?**

Arthritis is a highly prevalent health condition with an increasing negative impact. Nearly 1 in 4 adults in the United States (54.4 million persons) report having a diagnosis of arthritis, and the prevalence of arthritis-attributable activity limitation has increased 20% from 35.9% in 2002 to 42.8% in 2015.

**What is added by this report?**

In rural areas, arthritis affects nearly 1 in 3 adults. Rural residents with arthritis are likely to be limited by their arthritis, with approximately half reporting arthritis-attributable activity limitation. In rural areas, arthritis prevalence followed patterns previously reported for all adults with arthritis: higher prevalence among women, older adults, smokers, adults with less education, adults who are less physically active, or adults with higher body mass index.

**What are the implications for public health practice?**

Because of the high prevalence of arthritis in the rural adult population, rural residents should be targeted for interventions including physical activity and self-management education programs that help adults with arthritis manage their condition and reduce symptoms. Health care providers and community organizations can help residents participate in these helpful interventions.

## Footnotes

*
https://www.cdc.gov/brfss/annual_data/2015/2015_responserates.html .

†
https://www.cdc.gov/nchs/data/series/sr_02/sr02_166.pdf .

§
https://www.cdc.gov/nchs/data/statnt/statnt20.pdf .

¶
https://www.cdc.gov/arthritis/interventions/index.htm .

** https://acr.confex.com/acr/2007/webprogram/Paper7677.html.

††
http://askjan.org .

§§
https://www.cdc.gov/brfss/annual_data/2015/pdf/weighting_the-data_webpage_content.pdf .
